# Accurate Indoor Sound Level Measurement on a Low-Power and Low-Cost Wireless Sensor Node

**DOI:** 10.3390/s18072351

**Published:** 2018-07-19

**Authors:** Vladimir Risojević, Robert Rozman, Ratko Pilipović, Rok Češnovar, Patricio Bulić

**Affiliations:** 1 Faculty of Electrical Engineering, University of Banja Luka, 78000 Banja Luka, Bosnia and Herzegovina; vladimir.risojevic@etf.unibl.org; 2 Faculty of Computer and Information Science, University of Ljubljana, 1000 Ljubljana, Slovenia; robert.rozman@fri.uni-lj.si (R.R.); ratko.pilipovic@fri.uni-lj.si (R.P.); rok.cesnovar@fri.uni-lj.si (R.Č.)

**Keywords:** environmental noise monitoring, noise sensing, A-weighting, hardware platform, wireless sensor network

## Abstract

Wireless sensor networks can provide a cheap and flexible infrastructure to support the measurement of noise pollution. However, the processing of the gathered data is challenging to implement on resource-constrained nodes, because each node has its own limited power supply, low-performance and low-power micro-controller unit and other limited processing resources, as well as limited amount of memory. We propose a sensor node for monitoring of indoor ambient noise. The sensor node is based on a hardware platform with limited computational resources and utilizes several simplifications to approximate more complex and costly signal processing stage. Furthermore, to reduce the communication between the sensor node and a sink node, as well as the power consumed by the IEEE 802.15.4 (ZigBee) transceiver, we perform digital A-weighting filtering and non-calibrated calculation of the sound pressure level on the node. According to experimental results, the proposed sound level meter can accurately measure the noise levels of up to 100 dB, with the mean difference of less than 2 dB compared to Class 1 sound level meter. The proposed device can continuously monitor indoor noise for several days. Despite the limitations of the used hardware platform, the presented node is a promising low-cost and low-power solution for indoor ambient noise monitoring.

## 1. Introduction

Noise pollution is a common problem in urban environments. Typical environmental or background noise levels in residential areas range from 30 dB to 80 dB. It has been shown that noise pollution affects people’s health and cognition and long-term exposure to sound levels over 85 dB causes hearing damage [1]. Studies [2,3] investigated effects of exposure to office noise on comfort, symptoms, work performance and showed that everyday exposure to noise disturbance affected negatively comfort, selected symptoms, and self-assessed work performance. Participants were also less likely to make ergonomic, postural adjustments on their computer workstations while working in noisy conditions. The studies also showed that noise may even affect their judgment of the noise exposure.

Noise sensing and measurement are expensive due to the costs of measuring equipment and professional personnel. Wireless Sensor Networks (WSN) represent a promising and ubiquitous alternative to the current expensive noise sensing equipment and complex noise data collection procedures. Each node in WSN could measure the noise level at its location and the data would be collected by a sink node and forwarded to a web-based database. A noise-sensing node has to regularly sample the microphone’s signal at high frequency, an operation that consumes a significant amount of energy for an extended period of time. Consequently, we have two possibilities for further processing:

1. the node could transmit raw data to the sink, which is usually not feasible due to the memory, energy and network requirements for storing, sending and forwarding all these packets,
2. the node could process the data and transmit only the value of the sound level to the sink node.

To measure human exposure to noise, A-weighting filters are required. A-weighting is applied to the sound samples in an effort to estimate the loudness perceived by the human ear [1]. It is defined in the International standard IEC 61672-1 [4], which describes A-weighting filters, but does not define their implementation details. Most of the previous work [5,6,7,8,9,10,11,12] on noise measurement, has been done using an analog A-weighting filter. Usually, such a filter consists of five active stages implemented with operational amplifiers, and requires a significant amount of power.

The main goal of this work is to develop a sensor node for monitoring of indoor ambient sound levels. The sensor network, comprising a few stand-alone and battery-powered sensor nodes placed in different locations, gives us an assessment of sound levels in our home, school or office and helps us to better understand how ambient sound level affects our health, comfort and productivity. The key objectives in the design of a sensor node are cost, battery life and physical size. The node should continuously monitor sound and possibly other ambient parameters for several days without being plugged in the power socket. The key goals for the presented sound level meter are:

- implementation of A-weighting filtering on the fly with the magnitude response that satisfies the tolerance limits imposed by IEC 61672-1 standard,
- ability to measure the dynamic range of at least 60 dB and the sound pressure level up to 100 dB,
- low power consumption and price under 50 EUR.

As a solution to aforementioned goals, we present an accurate sound-level meter, which is a part of the low-power and low-cost wireless sensor node. Therefore, it is based on the ARM Cortex-M0—the smallest and the cheapest ARM processor core. For wireless communication with a gateway (sink) node, we use IEEE 802.15.4 compatible transceiver and the ZigBee protocol. As the node has limited processing abilities, we propose several simplifications to approximate costly signal processing stage and still accurately measure the sound level at the node. Furthermore, to reduce the communication between the sensor node and the sink node, as well as the power consumed by the ZigBee transceiver, we perform digital A-weighting filtering and non-calibrated calculation of the sound pressure level on the node itself, and consequently, send only the final value to the sink.

This paper presents the following primary contributions:

- implementation of an accurate indoor sound level meter on the smallest ARM core,
- magnitude response of the proposed A-weighting filter satisfies the tolerance limits imposed by IEC 61672-1 standard, despite the coefficient quantization,
- complete implementation of the proposed A-weighting filter on a sample-by-sample basis using only integer calculations,
- low power consumption of the implemented sensor node, which allows battery-powered operation for several days,
- mean difference between the proposed SLM and the Class 1 SLM is less than 2 dB on two experimental 12-min recordings.

Our work differs from the previous ones since our goal is to measure indoor sound level on a small, low-cost and low-power wireless sensor node with limited computational resources. Therefore, we propose a hardware platform for noise assessment based on the wireless sensor node and address the corresponding signal processing challenges. In addition, we have studied and implemented necessary signal processing algorithms required to perform the calculations. The implementation of signal processing algorithms has been adapted to the proposed hardware platform with limited computational resources. To further present the details about our work, we provide all the necessary equations and the resulting quantized filter coefficients. We also propose several simplifications to approximate a more complex and costly signal processing stage in order to assess the sound level with best possible accuracy.

This paper is organized as follows. Section 2 discusses related work. Section 3 provides sound level measurements basics and introduces the proposed digital A-weighting filter. In Section 4, the design and implementation of a low-power and low-cost sensor node for accurate sound-level measurement are described. Experimental results and calibration of the proposed device for sound level measurement are summarized in Section 5. Finally, the paper is concluded in Section 6.

## 2. Related Work

As already mentioned, one of the most promising technologies in the field of ambient sensing are Wireless Sensor Networks (WSN). Wireless sensor networks allow for accurate measuring of environmental pollution, spatially and temporally, and distributing this information to the public [13]. Furthermore, Dauwe et al. [14] present a multi-layered middleware platform for sensor networks, which offers data aggregation, control and management mechanisms. Di Francesco et al. in [15] provide a comprehensive taxonomy of the WSN architectures, present an overview of data collection process, and identify corresponding issues and challenges. Their research also provides an extensive survey of the related literature and hints to open problems and future research directions. Reis et al. [16] have outlined several developments within environmental sensing that offer new opportunities for data-intensive modeling for environmental and human health.

Wireless sensor networks for noise pollution monitoring are presented by Santini et al. [8] and Filipponi et al. [9], but these solutions do not perform signal processing in the sensor nodes. Instead they use dedicated hardware for noise measurements. However, no information has been given about implementation details. In addition, Wilson and Jarvis [10] describe a WSN for noise measurement which uses energy harvesting, but does not use frequency weighting. On the other side, Hakala et al. [5] and Kivelä et al. [6,7] present a design of a WSN for acoustic noise measurement in more details. However, in order to reduce the computational complexity, an analog A-weighting filter is used. The authors’ opinion is that a digital filter involves excessive floating-point calculations, which definitely surpasses the limit of a small of-the-shelf integer-based MCU. Consequently, they implement A-weighting filter with a cascade of three analog high-pass filters plus two analog low-pass filters.

Segura-Garcia et al. in [11] evaluate Tmote-Invent and Raspberry Pi nodes for urban noise pollution monitoring. The proposed nodes record the sound sequence, which prevents the implementation of the sound-level measurement on small low-power processors. Besides that, the study in [11] does not give more details on the filter or signal processing procedure used. The further work of Segura-Garcia et al. in [12] describes a distributed noise monitoring system and the practical application of a geo-statistical methodology for statistical spatial-temporal prediction of noise levels in semi-open areas, such as in a typical, small Mediterranean city. The authors in [12] also developed geo-statistical time model that allows the estimation of specific noise levels and characterization of the spatial-temporal variation of the noise pollution. The results confirm usability of the model as a good approximation of the experimental measurements.

Recently, mobiles phones emerged as a potential solution for the participatory noise monitoring; this approach was proposed by D’Hondt et al. in [17] and Kanjo in [18]. However, processing capabilities and power consumption of smartphones far exceed that of sensor nodes, which does not make them a viable solution for long-term noise pollution monitoring. However, ad-hoc short-term participatory measurements can be easily performed, but first we need to evaluate accuracy and efficiency of these platforms. Kardous and Shaw in [19] report on the accuracy of smartphone sound measurement applications on both the Android and the iOS operating systems. The authors generated pink noise in 20 Hz to 20 kHz frequency range, at levels from 65 to 95 dB in 5 dB increments. The study showed that certain sound measurement applications for Apple smartphones and tablets may be considered accurate and reliable enough to be used to assess noise levels, while Android and Windows applications were at that moment not accurate enough for noise assessments. Nast et al. in [20] compared the accuracy of smartphone applications used to asses the band-limited noise level to measurements made using a calibrated sound-level meter. This study showed that most mobile applications reported higher sound levels.

In the most recent study, Zamora et al. in [21] proposed the accurate noise assessment procedure using smartphones. They focused on the sound capture and processing procedure, analyzing the impact of different noise calculation parameters and algorithms. They have performed a series of experiments to determine the optimal procedure in term of accuracy when compared to a professional noise measurement unit. The authors implemented and compared three different algorithms used to obtain noise levels with A-weighting, showing that both the sampling rate and the selected buffer size can have a significant impact on the accuracy of noise level measurement. The obtained experimental results show that it is possible to measure noise levels using smartphones with the accuracy comparable to professional devices.

The study in [22] by Alsina-Pages et al. describes the possible design of an acoustic low-cost sensor network installed on public buses to measure the traffic noise in the city in real time. The authors do not provide any implementation details, but they plan to implement the sensor network for noise measurement in the near future. Before the implementation they address several most important challenges: (a) signal processing algorithms; (b) the bus engine noise and its contribution to the noise map; (c) the selection of the best bus routes for the measurements and the proper creation of the noise maps in the cloud from ubiquitous acoustic data; and finally, (d) the selection of the appropriate low-cost and low-power consumption platform, but with enough computational capacity to implement the necessary signal processing and communication algorithms to perform the measurements and afterwards send them to the cloud.

Noriega-Linares and Ruiz in [23] describe the prototype of an acoustic sensor based on the Raspberry Pi platform for the analysis of the sound field. The device is also connected to the cloud to share results in real time. The computation resources of the Raspberry Pi allow sampling the high quality audio for calculation of various acoustic parameters. The Raspberry Pi has a powerful computing core—as such it cannot be considered as a low-power platform. Therefore, it is less appropriate for a low-cost and low-power sensor node with limited power supply. The pilot test in this study has also served as an indication tool for the inhabitants of the neighborhood to be more aware of the noise in their environment.

Mydlarz et al. in [24] present the design and experimental measurements using a low-cost MEMS microphone. The measurements performed in this study show that analog MEMS microphone solution is suitable for accurate urban acoustic monitoring. The sensing device is based on the Tronsmart MK908ii board running Ubuntu Linux. In the proposed prototype system the Knowles SPU0410LR5H-QB analog MEMS microphone is used. The microphone has a flat frequency response between 100 Hz and 10 kHz. The device is used to monitor outdoor urban noise in the city of New York. The proposed device utilizes a constant connection to a 120 V mains supply via a domestic power outlet. This work also proposes three general categories, according to sensor functionality and cost: dedicated monitoring stations, moderately scalable sensor networks, and low-cost sensor networks. The presented solution in this article belongs to the third category, which typically consists of custom made nodes designed to be inexpensive, low-power and autonomous for large scale deployments. The majority of these devices utilize low-power single board computing cores and low-cost audio hardware with an effective range from 55 to 100 dB at 3 dB accuracy when compared to a Class 1 sound level meter. The price point of 150 USD per sensor node in this category makes it a viable solution for pervasive network deployments.

Blythe et al. in [25] presented the MESSAGE project and introduced a sensor node prototype for temporal and spatial urban (traffic) monitoring. The node includes a low-power PIC18F4620 MCU, a GPS module, a 3-axis accelerometer, a digital temperature sensor, a chemical sensor, a traffic sensor and an acoustic noise sensor. The sensor uses a low power Zigbee radio for communication with gateway nodes, which transfer data to a server. Unfortunately, the paper lacks any further details about the acoustic noise sensor part; however, the authors reported that good agreement (within 3 dB) was verified in the range 55–100 dB. Much more details about the same acoustic sensor node can be found in [26] by Bell and Galatioto. In addition, the authors present several practical deployments of up to 50 sensors (called “motes”) that were used to implement pervasive networks with the aim to improve the understanding of noise in urban environments and street canyons.

The study [27] aimed at getting the overall impression of the acoustic climate of living rooms in nursing homes. There is evidence that too loud sound-scape quality more generally can affect negatively the quality of life of people with dementia and increase agitation. The study aims to use the sound-scape approach to enhance the quality of life as well as improving the everyday experience of nursing homes. The sound levels were monitored during daytime in nine living rooms in five nursing homes, but the authors did not mention which sensor nodes were used in their project. Nevertheless, we believe that the device presented in this paper can be effectively used in sound level monitoring in (nursing) homes and can help to enhance the quality of life and working conditions.

## 3. Sound Level Measurement Basics

The human auditory system responds to air pressure changes, which are perceived as sound. Therefore, in order to quantify the sound level, it is convenient to measure the pressure of the sound wave at the location of the listener. The sound pressure level is computed as the root-mean-square (RMS) value of the sound pressure, $p_{RMS}$, relative to the reference pressure $p_{0}=20\;\mu \mathrm{Pa}$ and expressed in decibels [28]:

(1) $$SPL=20\log\frac{p_{RMS}}{p_{0}}\;\left[\mathrm{dB}\right].$$

Reference value $p_{0}$ is chosen to be approximately the threshold of hearing at 1000 Hz, for a typical human ear. The effective sound pressure is the RMS value of the instantaneous sound pressure *p* over a given interval of time. The RMS value of the sound pressure is defined as

(2) $$p_{RMS}=\sqrt{\frac{1}{T}\int_{0}^{T}p^{2}\left(\tau \right)d\tau }.$$

Since root mean square computation in Equation (2) involves time averaging, three values for the time constant *T* are adopted in sound level measurements, namely: impulse (I), fast (F), and slow (S) averaging with time constants equal to 35 ms, 125 ms, and 1 s, respectively [28]. Analog sound level meters use exponential averaging with the above time constants. On the other hand, digital sound level meters usually use linear averaging with integration times twice as long as the corresponding time constants in the analog case [29]. In this paper, a digital sound level meter design with fast (F) time linear averaging is presented. Therefore, the integration time is 250 ms. This value is chosen in order to enable monitoring of temporal variations of the sound level being measured.

The sound pressure level is an objective measure of the sound level. However, humans perceive sounds in a subjective manner and perceived sound loudness depends on the sound level, frequency bandwidth, spectral content, information content, as well as the duration of exposure to the sound. Therefore, it is considerably harder to quantify. Basics of loudness measurements have been established in the work of Fletcher and Munson [30]. Studying the relationship between sound level and loudness for tones of various pitches, Fletcher and Munson came to the set of equal-loudness contours showing the intensities of tones of different frequencies that are perceived to be equally loud by the average listener. Acoustical Society of America developed the first standard for sound level meters (American tentative standards for sound level meters for measurement of noise and other sounds Z24.3-1936) based on the Fletcher and Munson curves. To take into account the impact of frequency to human perception of loudness, the IEC 61672-1 standard [4] for sound-level meters defines three types of frequency weighting: A, C, and Z. The frequency weighting A is mandated for all sound-level meters, C-weighting is required for sound level meters conforming to class 1 tolerance limits, and Z-weighting is optional.

Although there is criticism that A-weighting is not well correlated with human perception of loudness [31], it is legally required in many countries and enables comparison of measurement results with historical data. Therefore, it is the frequency weighting adopted for sound level meter design presented in this paper. However, it should be noted that the other frequency weightings could be easily implemented following the described approach.

**Figure 1:**
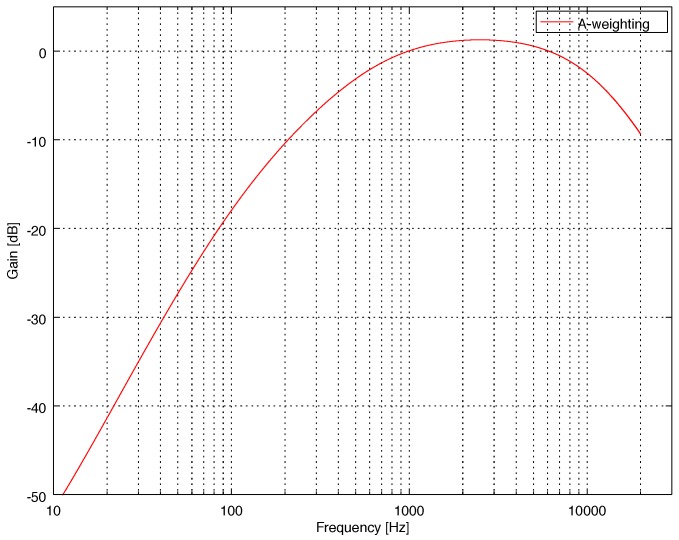
Magnitude response of the A-weighting filter.

The A-weighting filter [1,4] is a bandpass filter whose frequency response (Figure 1), defined for frequencies in the audible range (20 Hz to 20 kHz), emphasizes the frequency band from 1 to 4 kHz since humans are most sensitive to noise-induced hearing loss in that range. The transfer function of an analog A-weighting filter is defined in [4] as

(3) $$H_{a}\left(s\right)=\frac{4\cdot \pi^{2}\cdot 12194^{2}\cdot s^{4}}{{\left(s+2\pi \cdot 20.6\right)}^{2}{\left(s+2\pi \cdot 12194\right)}^{2}\left(s+2\pi \cdot 107.7\right)\left(s+2\pi \cdot 739.9\right)}$$

After the measured signal has been filtered with the A-weighting filter defined in (3), its root mean square value is computed and the obtained value of sound level is reported in decibels, denoted as dBA.

## 4. Implementation Details

The proposed sound Level Meter has been designed and implemented on a general wireless sensor node as a basis. One of the main challenges was the implementation of A-weighting filter on such resource limited system with fixed-point calculation unit. As an additional challenge, various details have been addressed to perform proper sound level calculations regarding all existing limitations. All those details are presented in continuation of this section.

### 4.1. Wireless Sensor Node

**Figure 2:**
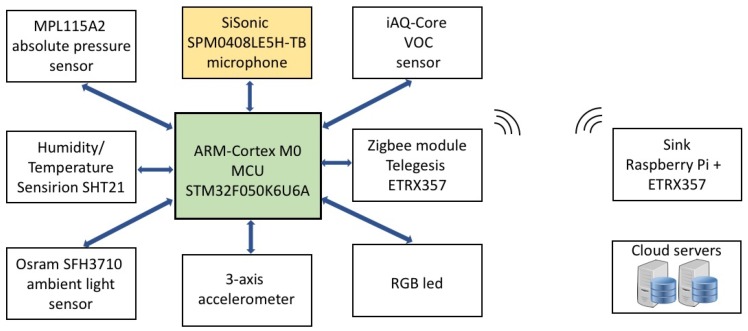
Architecture of the wireless sensor node.

The sound level meter described in this paper is a part of the low-power and low-cost wireless sensor node used in a wireless sensor network for monitoring ambient conditions. Figure 2 shows the architecture of the wireless sensor network, which consists of sensor nodes and the sink node. Wireless sensor nodes are based on the ARM Cortex^TM^-M0 MCU—the smallest ARM core, while the sink node is the Raspberry Pi computer. Nodes communicate with each other and with the sink node via ZigBee. Each node senses sound pressure level and other environmental parameters and sends the processed data to the sink node, which formats data and sends them to the server.

**Figure 3:**
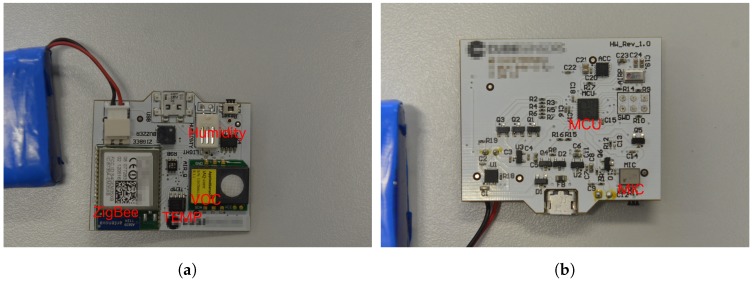
The sensor node. (**a**) The top side of the node’s PCB. It contains the humidity sensor, the VOC sensor, the temperature sensor and the ZigBee transceiver. (**b**) The bottom side of the node’s PCB. It contains the ARM Cortex^TM^-M0 MCU and the SiSonic microphone.

The wireless sensor nodes are powered by 3.7 V Lithium Polymer (LiPo) batteries with capacity of 2900 mAh. They are built around STM32F050K6U6A MCU with UFQFPN32 32-pin package pinout and ARM 32-bit Cortex^TM^-M0 MCU with the clock frequency of 48 MHz. MCU has 32 Kbytes of Flash memory and 4 Kbytes of SRAM. Each node also contains a MEMS microphone, as well as a humidity and temperature sensor, an absolute pressure sensor, an ambient light sensor, a sensor used to measure VOC (volatile organic compounds) levels and provide CO_2_ equivalent and total VOC equivalent predictions, and a 3-axis accelerometer used to detect shake. The node uses the Telegesis ETRX357 module for ZigBee communication and has an RGB LED used to signal different activities and states of the sensor node. The sensor node is shown in Figure 3a (front) and Figure 3b (back) and the Table 1 shows the list of components and corresponding costs for the prototype sensor node.

According to [24] acoustic sensors can be grouped into three general categories, where sensor functionality and cost are the focus. The category 3 of sensor network typically consists of custom made nodes designed to be inexpensive, low-power and autonomous for large-scale deployments. The majority of these utilize low-power single board computing cores with low-cost audio hardware. The price point of 150 USD per sensor node in this category makes it a viable solution for pervasive network deployments. Comparing to the price of the nodes form [24] (81 USD) and [5] (100 EUR), this node is very competitive.

The sensor nodes can be placed in any room or office to continuously measure noise. The node sends data to the server so the user can access it remotely. The noise monitoring can help user to reduce stress, sleep better, work smarter or be a more responsible neighbor. The Zigbee based sensor network allows for monitoring several rooms simultaneously.

#### 4.1.1. The Microphone

**Figure 4:**
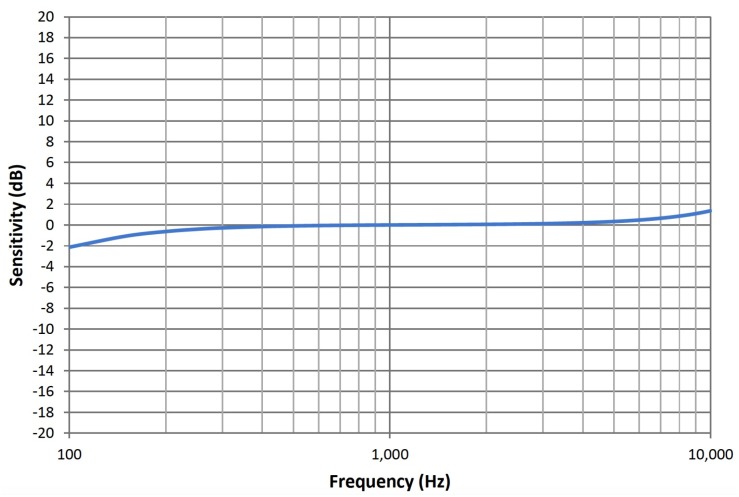
Typical free field frequency response of the SiSonic microphone normalized to 1 kHz [32].

The sensor node uses the SiSonic SPM0408LE5H-TB [32] low-power MEMS microphone to measure the sound level. SiSonic SPM0408LE5H-TB is a miniature, high-performance, low power, bottom port silicon microphone. The bottom-port package, where the sound inlet is placed in the bottom, optimizes the acoustic performance of the microphone in terms of SNR and also allows obtaining a flat response across the entire audio band. The SPM0408LE5H-TB microphone consists of an acoustic sensor, a low noise input buffer, and an output amplifier. This device is suitable for applications such as sensors and other portable electronic devices, where excellent wide-band audio performance and RF immunity are required. According to [32], the typical sensitivity at 1 kHz is −18 dBV/Pa. Its frequency response curve is depicted in Figure 4. The frequency range of SiSonic SPM0408LE5H-TB fits well into A-weighting frequency curve. As the microphone has a flat frequency response, it is suitable when natural sound and high intelligibility of the system is required.

Although the response of the microphone exhibits very little variation over its operating range, placement of the microphone on the PCB may introduce changes to this response. The microphone requires a path for the sound through the bottom port and a hole in the PCB is required to transfer sound waves into the microphone package. Due to the small size of the microphone packages and their related features, the exact geometry of the sound path does not significantly influence the response of the microphone. According to [32], the response of a MEMS microphone is not affected by PCB thickness or the PCB hole size, as long as the hole is not smaller than 0.25 mm in diameter. A 1 mm diameter for the hole is used. Also, the sensitivity of MEMS microphones varies very little over temperature, a fraction of a decibel at most [32]. However, Mydlarz et al. in [24] used sine sweep method to create the impulse response of the MEMS microphone mounted on the PCB. They showed that when the microphone is mounted on the PCB, unwanted effects and resonance (i.e., the peaks and troughs in sensitivity) may develop in the frequency range between 2 and 20 kHz. Also, the rise in response after 10 kHz may develop as a result of the Helmholtz resonance created by the microphone’s inner chamber and PCB port. To compensate for the MEMS microphone response in real-time, they implemented an inverse linear-phase FIR filter for the time-domain filtering. The FIR filter required 8192 coefficients to match the desired response and was implemented using the optimized DSP routines of the mini PC’s Cortex A9 processor.

Taking into account these technical microphone’s features and a large number of inverse filter coefficients, we concluded that a costly inverse filter of the microphone’s frequency response cannot be implemented on a system with limited resources, and it is not necessary for the Category 3 device, as classified in [24]. The calibration of the final sound level meter is sufficient and this has been also confirmed by the results in Section 5.

#### 4.1.2. Communication

The sensor network uses license-free, low-power IEEE 802.15.4-based communication standard, known as Zigbee, to provide power-efficient networking over ranges of up to 100 m. Zigbee is an open standard that provides wireless networking in the 2.4 GHz band at data rates up to 250 kbps. Its main advantages are low power consumption and small antenna dimensions.

The presented node uses the Telegesis ETRX357 module for wireless communication. It is a low-power Zigbee communication module that includes a 2.4 GHz, IEEE 802.15.4 compliant transceiver. The integrated receive channel filtering feature allows robust co-existence with other communication standards in the 2.4 GHz frequency band, such as IEEE 802.11 and Bluetooth. Consequently, this module is suitable for indoor use where other communication devices are also present. As the ETRX357 module represents a complete embedded system-on-chip solution, its behavior can be controlled externally through unique AT-style command serial line interface. Therefore, it can be quickly integrated into the sensor node application without the need for RF expertise and complex software engineering. Typical consumption of these modules is 25–30 mA at +3 dBm output power.

ZigBee standard comprises two types of nodes: Full Function Device (FFD) and Reduced Function Device (RFD). The latter type is intended for low-power sensor nodes such as our proposed solution. Only a subset of network functions is implemented in these nodes and consequently, they can be realized with simpler, low-power embedded platforms. Typically, RFD nodes are active only a fraction of total time, the majority of the time they are put into a low-power sleep mode. The FFD nodes perform additional network coordination, routing functions and are usually not battery powered.

In the proposed system, the sink node is always powered on and operates as an FFD device. On the other hand, the sensor node acts as an RFD device, when it is powered by the battery. However, when the sensor node is connected to external USB power supply, the battery is charged and at the same time, the node reconnects to the network as an FFD device and performs additional routing functions. In this case, it can extend the range and help to connect more distant sensor (RFD) nodes to the network. Therefore, the user can easily extend network range by keeping certain nodes on permanent USB power supply. As soon as USB power supply is disconnected, the node reconnects to the network as an ordinary RFD device and continues sensing operation.

As described earlier, the sensor node acquires samples of the sound signal, performs local processing and sends final results to the sink node connected to hard-wired power. The sink node processes the data on the higher level and transfers outcomes via WiFi connection to a remote server, where they are thoroughly analyzed and presented to end users via a web interface. In the opposite way, the user or server can send commands to each sensor node from the remote server as well. Commands are always routed through the sink node since sensor nodes are sleeping most of the time. In addition, the sink node can also generate commands for sensor nodes. The actual transfer in the opposite direction is performed only when sensor nodes are active (non-sleeping) and communicating with the sink node.

To illustrate the explanation, let us present the real case example. The sensor node in the living room collects values from on-board sensors and sends them to the server via the sink node. The sink node and server application can further process and analyze the data from this node. For instance, if some extreme values are observed (e.g., high sound pressure level), the server sends the “glove LED” command to the sensor node in the living room via the corresponding sink node. The sensor node then operates the RGB LED according to the received command. In this particular example, RGB LED is softly switched on in red color and signals the user that some action must be performed.

Firmware for sensor and sink nodes was designed with the main goal of network stability and fault tolerance. Various fault events were predicted and taken into account during the design of the main loop flow. All nodes continuously monitor network connection and act upon any potentially harmful event (e.g., disconnection, external noise, change of frequency channel). The main goal for sensor nodes is their low power consumption. Therefore, most of the time, sensor nodes are in low-power sleeping mode; they wake up periodically on timer event, acquire sensors’ states, process and send the data to the sink node, wait for confirmation or command from the sink node and then return into a sleep mode again. The sleep period along with other parameters can be sent to the node from the server via the sink node. Using this approach, an efficient compromise between node’s power consumption and sensing rate can be determined for each specific application or even adapted dynamically during the system’s operation.

### 4.2. Design of a Digital A-Weighting Filter

**Figure 5:**
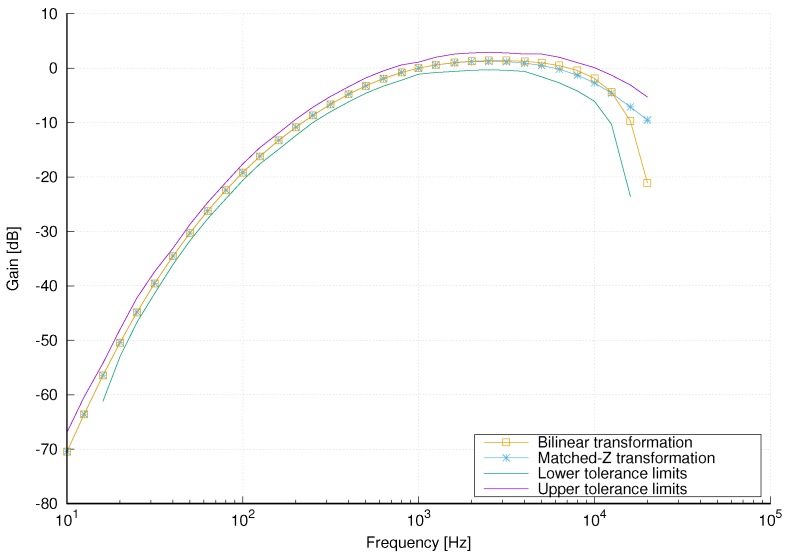
Magnitude responses of digital A-weighting filters designed using bilinear and matched-z transformations. Tolerance limits for filters in Class 1 sound level meters are shown.

In this subsection we present the design and implementation of the proposed A-weighting filter that is used in the Sound Level Meter. The most common techniques for digital filter design from an analog filer are impulse invariance, bilinear transformation and matched-z transformation. Impulse invariance is not applicable to this case due to severe aliasing, whereas A-weighting filter design using bilinear transformation was discussed in [1]. For comparison, we designed filters using both bilinear and matched-z transformations. We observed that the magnitude response of the filter designed using matched-z transformation slightly violates the tolerance limits and, in order to correct the magnitude response and avoid possible errors due to coefficient quantization, we added another low-pass section. The magnitude responses of the filters designed using bilinear and matched-z transformations are shown in Figure 5.

**Figure 6:**
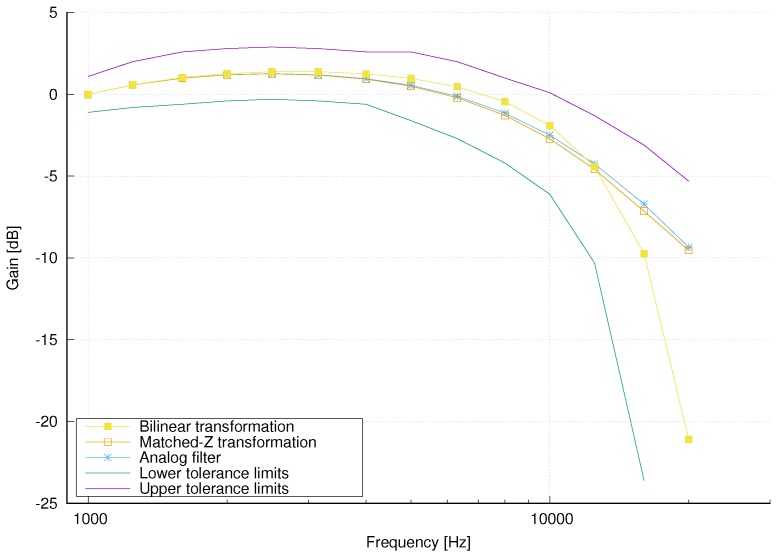
Enlarged portion of the magnitude responses of digital and analog A-weighting filters in the frequency range above 1 kHz.

It can be seen that both magnitude responses are within the tolerance limits for filters in Class 1 sound level meters. Moreover, for frequencies up to 2 kHz, they are almost identical to each other and to the magnitude response of the original analog filter. In Figure 6 an enlarged portion of the magnitude responses in the frequency range above 1 kHz is shown. It can also be seen that the magnitude response of the filter designed using matched-z transformation is slightly closer to the magnitude response of the analog filter than the magnitude response of the filter designed using bilinear transformation. Therefore, we have chosen the A-weighting filter designed using matched-z transformation for our implementation of the Sound Level Meter.

Using matched-z transformation for the transfer function given in Equation (3) of the analog A-weighting filter, and sampling frequency $F_{S}=48$ kHz, the transfer function of a digital filter is obtained:

(4) $$H_{d}\left(z\right)=\frac{{\left(1-z^{-1}\right)}^{4}}{{\left(1-0.9973z^{-1}\right)}^{2}{\left(1-0.2025z^{-1}\right)}^{2}\left(1-0.9860z^{-1}\right)\left(1-0.9097z^{-1}\right)}.$$

With the added low-pass section for correction of the magnitude response, the resulting transfer function is:

(5) $$H\left(z\right)=\frac{\left(1+0.3z^{-1}\right){\left(1-z^{-1}\right)}^{4}}{{\left(1-0.9973z^{-1}\right)}^{2}{\left(1-0.2025z^{-1}\right)}^{2}\left(1-0.9860z^{-1}\right)\left(1-0.9097z^{-1}\right)}$$

As can be seen from Equation (5), the obtained filter has poles in the vicinity of the unit circle and can become unstable due to the round-off errors in the fixed-point realization. Therefore, we have decided to use a cascade-form realization with the second order sections (SOS) as described in [33] using direct form II:

(6) $$H\left(z\right)=H_{1}\left(z\right)H_{2}\left(z\right)H_{3}\left(z\right).$$

Transfer functions of the SOS are:

(7) $$\begin{array}{c} \begin{array}{cc} H_{1}\left(z\right) & =\frac{1+0.3000z^{-1}}{1-0.4053z^{-1}+0.0411z^{-2}}\\ H_{2}\left(z\right) & =\frac{1-2.0000z^{-1}+1.0000z^{-2}}{1-1.8939z^{-1}+0.8952z^{-2}}\\ H_{3}\left(z\right) & =\frac{1-2.0000z^{-1}+1.0000z^{-2}}{1-1.9946z^{-1}+0.9946z^{-2}} \end{array} \end{array}$$

### 4.3. Implementation of the Sound Level Meter

**Figure 7:**
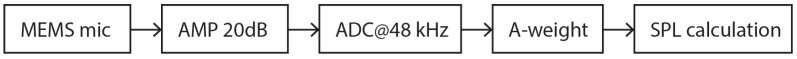
Block diagram of sensor’s SPL functionality.

Figure 7 shows the functionality of the implemented sound level meter. The sound is captured by a MEMS microphone, amplified and passed to ADC, which samples the input signal at 48 kHz. The digital samples are then passed to the digital A-weighting filter described in the previous subsection. In the last stage the non-calibrated sound pressure level ($SPL_{NC}$) is calculated according to Equation (8):

(8) $$SPL_{NC}=10\log p_{RMS}^{2},$$

where we implemented the computation of the RMS value, $p_{RMS}$, defined in Equation (2) by summing the squared values of N filtered sound samples y[n], where N is the number of samples corresponding to the chosen integration time:

(9) $$p_{RMS}^{2}=\frac{1}{N}\sum_{n=0}^{2}y^{2}\left[n\right].$$

The SPL result is then corrected according to the polynomial fitting used for the calibration as described in Section 5.

#### 4.3.1. Amplification Stage

**Figure 8:**
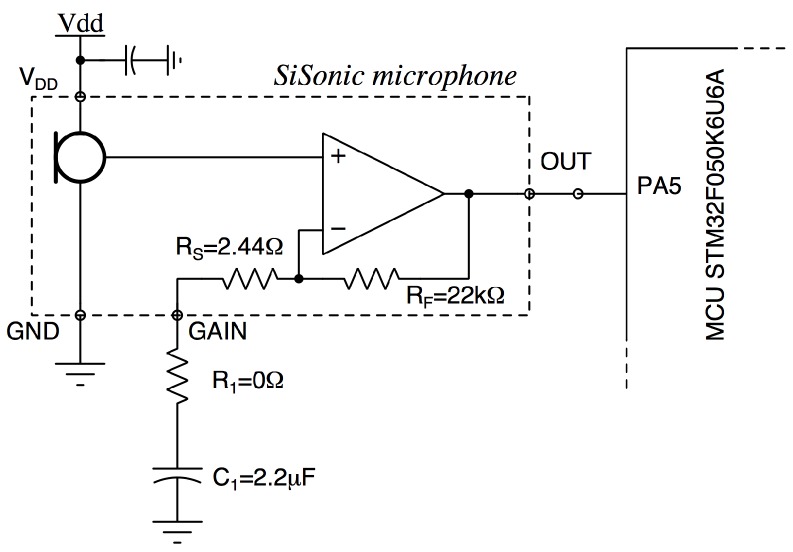
The interface circuit of the Sisonic microphone. The output is connected to the integrated 12-bit ADC (pin 5) of STM32F050K6U6A MCU.

In far-field applications like sound-level metering, the desired acoustic signal is in the microphone’s far-field and usually requires additional amplification. Amplified SiSonic microphones add up to 20 dB of gain to the analog output signal before transmitting it to the CPU. Amplifying the signal at the mic versus improves the overall system Signal-to-Noise Ratio (SNR) by increasing the transmitted signal size relative to noise in the traces. The voltage waveform from the acoustic sensor is pre-amplified using an (op-amp)-based active amplifier before being processed by the microcontroller’s analog to digital converter (ADC) as depicted in Figure 8. The value of R1 is chosen to give the desired gain value, with a maximum gain of 20 dB when R1 is 0. C1 is chosen so that the corner frequency of the high-pass filter formed by C1, R1, and RS is set to 30 Hz (i.e., well below the acoustic range).

#### 4.3.2. ADC Stage

The microphone is connected to the PA5 pin of MCU and fed to internal ADC. The STM32F050K6U6A MCU has a 12-bit resolution ADC that can compensate dynamic range of $20\log(2^{12}-1)=72$ dB. The sampling time is 250 ms at 48 kHz. The conversion formula for ADC used in STM32F050K6U6A MCU is as follows:

(10) $$V_{\mathrm{ADC}}=\frac{V_{\mathrm{DDA}}}{2^{12}-1}\times \mathrm{ADCDATA},$$

where $V_{\mathrm{DDA}}$ is input analog power supply voltage, which is 3.3 V. $V_{\mathrm{ADC}}$ is the voltage fed by microphone’s op-amp and is in the range of [0, 3.3] V and ADCDATA is the 12-bit binary representation of $V_{\mathrm{ADC}}$. Assuming that the input voltage $V_{\mathrm{ADC}}$ is a sinusoidal wave with the peak voltage of 3.3 V, the RMS voltage at the output of the microphone’s op-amp is $V_{\mathrm{RMS}}=3.3\;V/\sqrt{2}=2.33345$ V. As the op-amp has the gain of 20 dB, the microphone RMS voltage is 233,345 mV. Taking into account that typical microphone sensitivity is −18 dBV/Pa and that the gain of the op-amp is 20 dB, we can calculate the microphone sensitivity sen in V/Pa as:

$$sen=10^{\frac{-18}{20}}=125\;\mathrm{mV}/\mathrm{Pa}$$

Now, the maximum value of sound pressure level, $p_{RMS}$, that corresponds to $V_{\mathrm{RMS}}=2.33345$ V can be obtained as:

$$p_{RMS}=\frac{V_{RMS}}{sen}=1.87\;\mathrm{Pa}$$

Applying Equation (1), we can calculate that the proposed system is able to measure the sound pressure level up to:

$$SPL_{max}=20\log\frac{1.87}{20\cdot 10^{-6}}=100\;\mathrm{dB}.$$

#### 4.3.3. A-Weighting Stage and SPL Calculation

The digital A-weighting filter is implemented in the C language on the presented sensor node that has two major constraints:

1. ARM 32-bit Cortex^TM^-M0 MCU does not have the floating-point unit,
2. there is only 4 kB RAM memory, which prevents sound recording and imposes “on-the-fly” signal processing of the sound samples.

The node performs sampling of the sound, A-weighting filtering and calculates the sum of squares of all filtered samples. This value is then sent to the sink node, which calculates the sound pressure level. As the ARM 32-bit Cortex^TM^-M0 MCU does not have the floating-point unit, we have to implement the digital A-weighting filter using a suitable integer number format. Also, the digital A-weighting filtering should be performed on-the-fly, because there is not enough memory to store all the samples.

The filter is implemented as three cascaded SOS with transfer functions given in Equation (7). All SOS coefficients are quantized to 16 bits. Having in mind that we use a 12-bit ADC, and that we shift right each product by 16 bits, the maximum value that can be obtained from one filter section is represented with 14 bits. The maximum value obtained from three filter sections is represented by 16 bits. After squaring, the maximum value is represented by 32 bits. Finally, the window length of 250 ms at 48 kHz results in 12,000 samples and the sum of 12,000 squares requires 46 bits for its representation. To represent such a big number, we have decided to use 64-bit integer number format to represent Q48.16 fixed-point numbers. All samples from the microphone are first multiplied by $2^{16}$. After all filter calculations are performed, the result is multiplied by $2^{-16}$ and thus represented in the Q48.16 fixed-point format. The quantized filter coefficients for all three SOS of the A-weighting filter multiplied by $2^{16}$ are

(11) $$A=\left[\begin{array}{ccc} 65536 & -26543 & 2687\\ 65536 & -124120 & 58669\\ 65536 & -130719 & 65183 \end{array}\right]$$

and

(12) $$B=\left[\begin{array}{ccc} 65536 & 19660 & 0\\ 65536 & -131072 & 65535\\ 65536 & -131072 & 65536 \end{array}\right]$$

**Figure 9:**
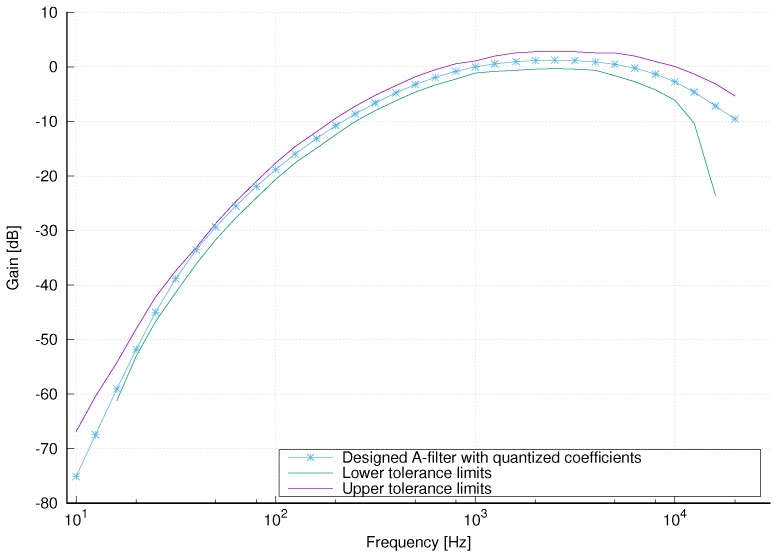
Magnitude response of the A-weighting filter with quantized coefficients.

To evaluate the influence of the coefficient quantization to the magnitude response of the filter, all SOS coefficients are multiplied with $2^{16}$, quantized to 64 bits and magnitude responses for all second order sections are computed. The magnitude response of the overall filter is shown in Figure 9 and satisfies the tolerance limits imposed by IEC 61672-1 standard.

The filtering is performed on the sensor node and the final sum of squares of the filtered samples, given by Equation (9), is sent to the sink, which performs the conversion of Q48.16 to floating-point and finally calculates the sound pressure level, according to Equations (8) and (13).

## 5. Results and Calibration

To investigate the suitability of the presented platform to be used as indoor noise pollution sensor, we deployed the sensor nodes in two different indoor locations and performed 12-min noise measurements sessions and compare it with the Type I sound meter. Before setting up the noise measurements sessions, we performed a calibration to avoid the misalignment in the measured parameters due to the mismatched microphones’ sensitivities and frequency responses. These results, along with detailed node power consumption, are described in the following subsections.

### 5.1. Calibration

**Figure 10:**
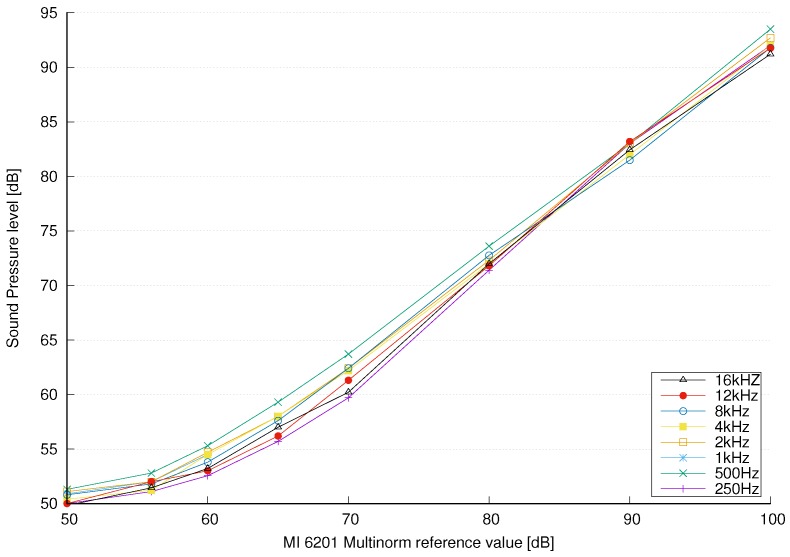
The results for various frequencies of sine waves obtained by the sensor. The horizontal axis represents the sound pressure levels obtained with the MI 6201 MULTINORM sound meter.

We have generated sound sine waves at 31.5 Hz, 63 Hz, 125 Hz, 250 Hz, 500 Hz, 1 kHz, 2 kHz, 4 kHz, 8 kHz, 12 kHz and 16 kHz. For each sound wave we set different sound pressure levels: 50 dB, 56 dB, 60 dB, 70 dB, 80 dB and 100 dB. These sound pressure levels were set with MI 6201 MULTINORM, using 125 ms time averaging. Then we measured sound pressure levels using the sensor node. The sensor node and its microphone pick-up point were very close to the MI 6201 MULTINORM sound meter, approximately 1 m away from the sound source. Figure 10 shows the non-calibrated SPL values for various frequencies of sound waves obtained with the sensor node versus MI 6201 MULTINORM references. The results show that the sound pressure levels were lower than sound pressure levels obtained with the MI 6201 MULTINORM sound meter. We can also observe a slight nonlinearity due to poor quality of the used microphone and overall acoustic transfer function of the sensor node and its housing.

**Figure 11:**
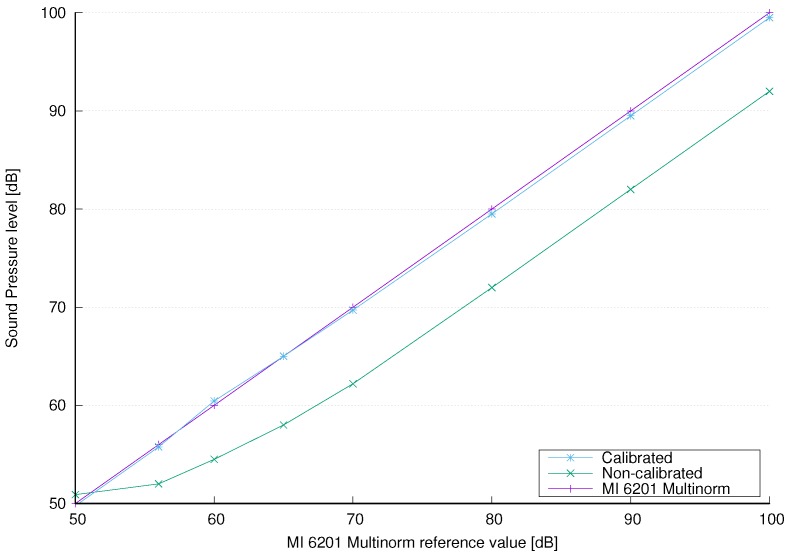
The calibration measurements and the fitting curve. The horizontal axis represents the sound pressure levels obtained with the MI 6201 MULTINORM sound meter, and the vertical axis represents the measured sound pressure levels before and after calibration.

To reduce the error we need to calibrate the sensor nodes. Calibration was done with MI 6201 MULTINORM, which is a universal all-in-one instrument for measurement of air temperature, air velocity, relative humidity, illuminance, luminance and includes Class 1 (Pro Set) sound level meter with two independent measuring channels compliant with IEC 61672 standard. Each channel can be set with different time and frequency weighting. The calibration acoustic source must be 1 kHz sine wave [4]. Using the measurement data from Figure 10 in the range of [50, 100] dB we obtained a polynomial fitting as:

(13) $$SPL=\left\{\begin{array}{cc} 2.65\cdot SPL_{NC}-83.52, & 50\leq SPL_{NC}<60\;\mathrm{dB}\\ 1.30\cdot SPL_{NC}-10.42, & 60\leq SPL_{NC}<70\;\mathrm{dB}\\ 1.01\cdot SPL_{NC}+7.49, & SPL_{NC}\geq 70\;\mathrm{dB} \end{array}\right.$$

where $SPL_{NC}$ is non-calibrated value of the sound pressure level obtained by the sensor node. The measured and calibrated SPL are depicted in Figure 11.

### 5.2. Indoor Accuracy Tests

**Figure 12:**
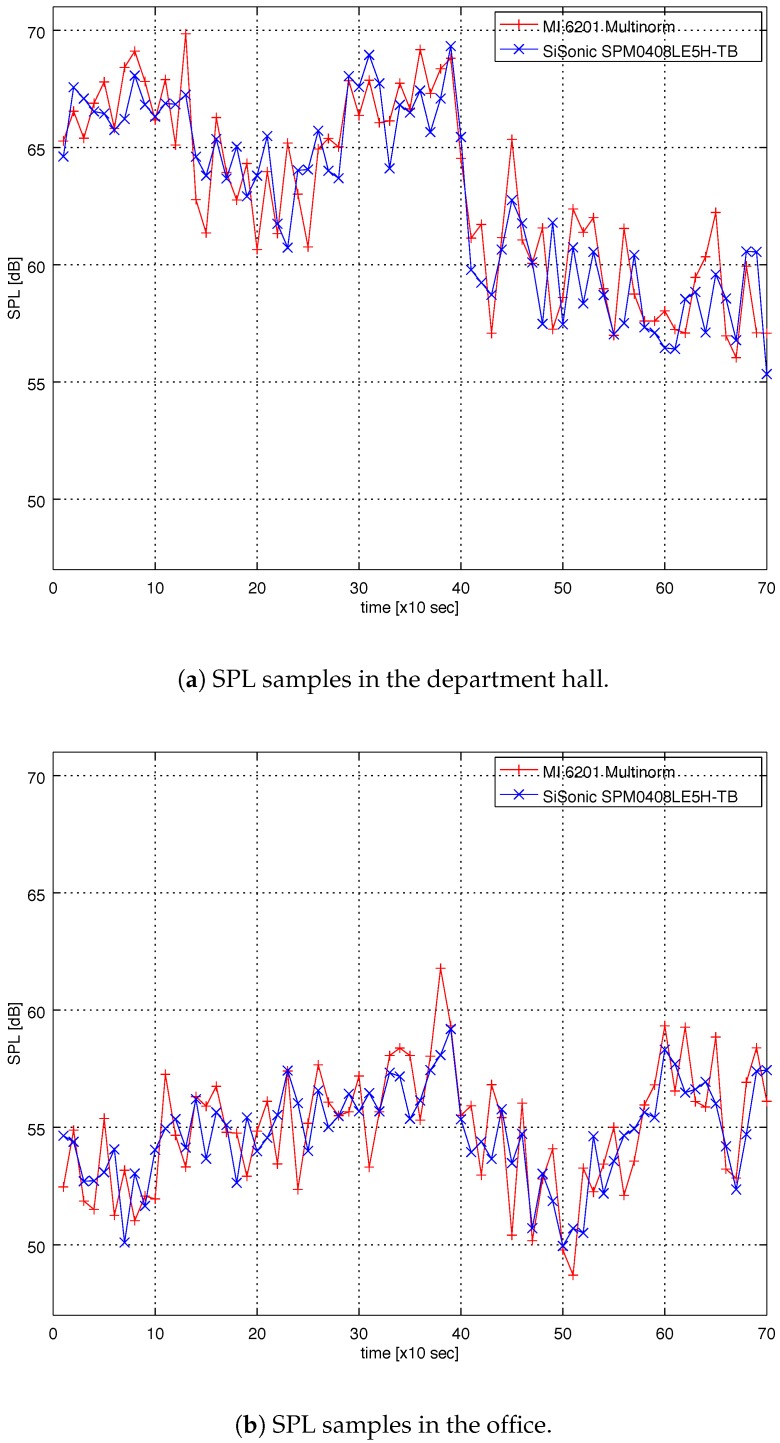
(**a**) SPL samples of recordings in the department hall during a break between lectures. At the time 400 s lectures resume and SPL drops; (**b**) SPL samples of recordings in the office with six people. Both recordings lasted 12 min, the samples are taken in 10 s intervals.

To assess the ability of the proposed sensor node to capture meaningful sound pressure level data, a 12-min indoor audio monitoring was conducted in two different indoor locations: the university department hall during a break between lectures, where a large number of students transfer between lecture rooms and hang out with each other, mainly during the pause between lectures; and the office at the university with six (6) researchers. Audio monitoring was conducted using both the proposed sensor node and MI 6201 MULTINORM in 10 s intervals. The history data collected from both devices are shown in Figure 12a (department hall) and Figure 12b (office).

Both recordings include numerous impulsive events (door closure, conversation, moving chairs, etc.). As can be seen from Figure 12a,b, the proposed sensor node closely follows the measurements made by MI 6201 MULTINORM. It should be emphasized that, although both sensors record samples in 10 s intervals, they do not necessarily record samples at the same time.

To assess the correlation between the measurements made with two different devices, the Pearson’s correlation coefficient was calculated for the 12 min interval. The value for the correlation coefficient is 0.88, which indicates a strong correlation between the signals. The mean difference between two devices for the both 12-min measurements is 1.6 dB.

**Figure 13:**
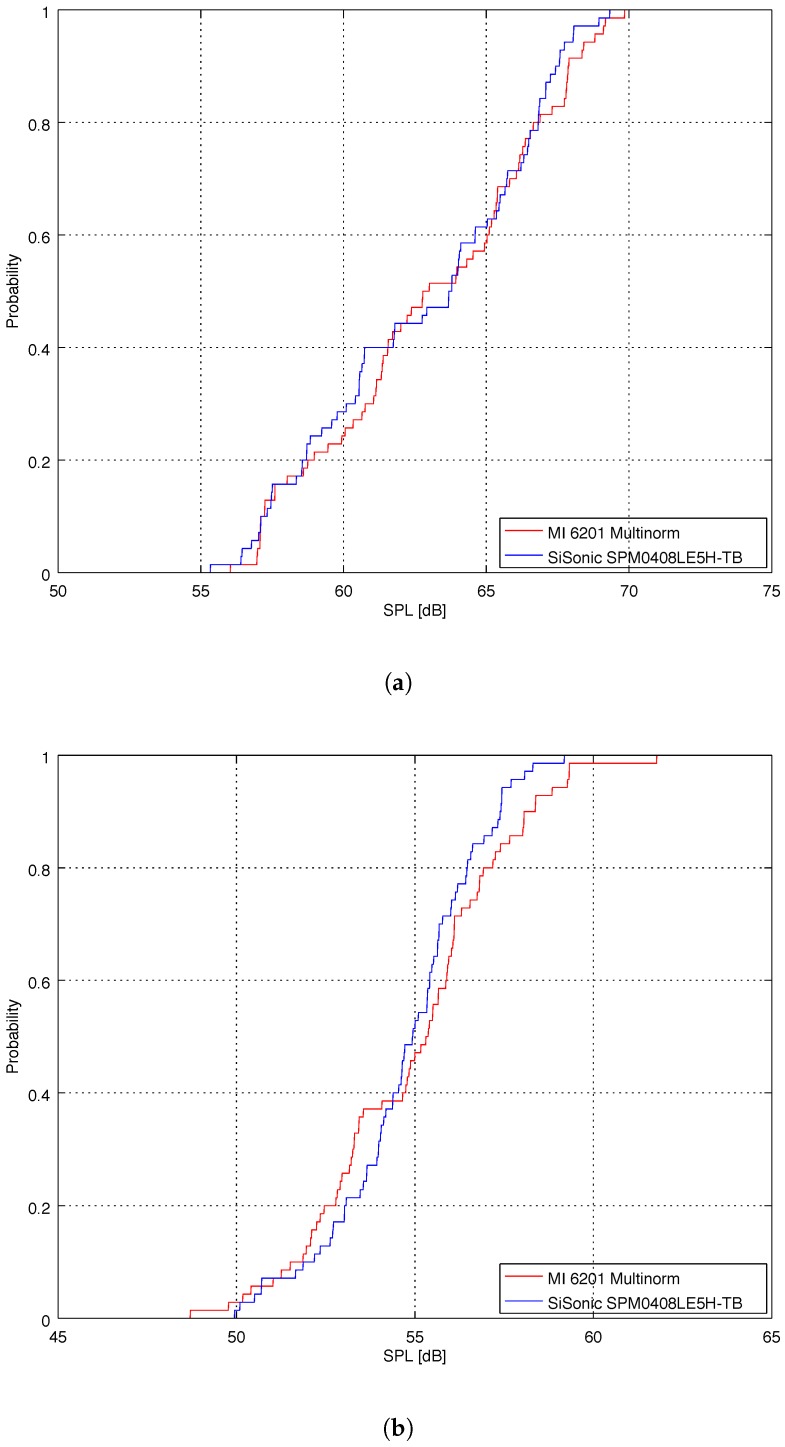
(**a**) Cumulative distribution function of SPL samples in the department hall; (**b**) Cumulative distribution function of SPL samples in the office with six people.

The cumulative distribution functions of samples are shown in Figure 13a (department hall) and Figure 13b (office). To compare cumulative distribution functions (CDF) of two empirical samples we have performed a 2-sample Kolmogorov-Smirnov test. The Kolmogorov-Smirnov test quantifies a distance between the empirical distribution functions of two samples. It returns a D statistic (the maximum vertical distance between the two empirical cumulative distribution functions) and a *p*-value corresponding to the D statistic. If the D statistic is small or the *p*-value is high, then we cannot reject the hypothesis that the distributions of the two samples are the same. Usually, we can reject the null hypothesis if the *p*-value is less than 0.05. In other words, for an identical distribution, we cannot reject the null hypothesis if the *p*-value is high. For the CDTs from Figure 13a we obtained D=0.11429 and high *p*-value = 0.75053. For the CDTs from Figure 13b we obtained D=0.14286 and high *p*-value = 0.47271. As both D statistic are small and both *p*-values are high, we cannot reject the hypothesis that the distributions are the same.

### 5.3. Power Consumption

Real-time power consumption was measured using a LeCroy WaveRunner 204xi, 2 GHz, 10 Gs/s oscilloscope. A sensor node and a 1 Ohm, 1% Manganin shunt resistor were connected in series and the voltage over this resistor was measured. The sensor node stays in sleep mode until it is awaken by the 3-axis accelerometer or the timer. The timer wakes the sensor node every 2.3 s to read the VOC sensor. Regular dummy reads of VOC sensors are required by sensor’s specifications. The sensor node is also awoken every 10 s (this time can be changed according to the application’s requirements) to read all sensors and to send data to the sink. The sensor node can be also awaken at any time by shaking it. In this case the sensor node reads all sensors and sends data to the sink.

**Figure 14:**
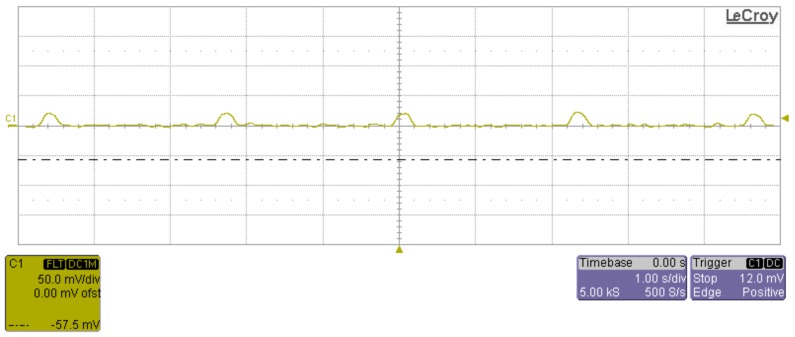
Oscilloscope record of power consumption (voltage over 1 Ohm resistor) during sleep mode.

The sensor node’s current consumption in sleep mode while regularly reading the VOC sensor is presented in Figure 14.

The peak current while reading the VOC sensor is 20.8 mA. Average power consumption in this operation is 2.7 mA.

**Figure 15:**
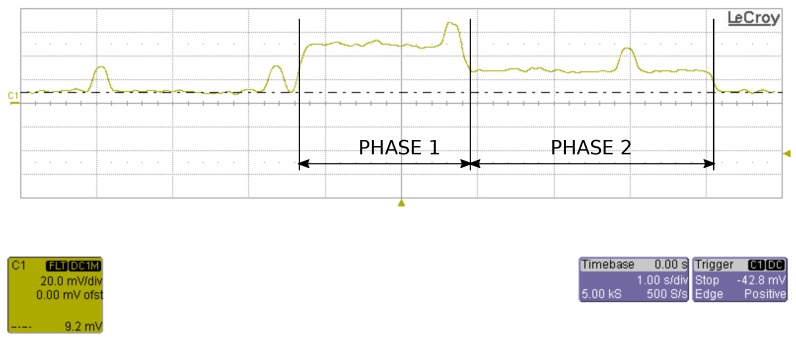
Oscilloscope record of power consumption (voltage over 1 Ohm resistor) during sensing and transmission.

The sensor node’s current consumption when reading all sensors and sending data to sink is presented in Figure 15. It includes two different phases. In the first phase the RGB LED glows for two seconds indicating that the sensor has been awaken. In the second phase RGB LED is switched off and it lasts for around 3 s (it depends on network communication). Immediately after awakening, all sensors are read, then the Zigbee radio is turned on and data is sent to sink. Reading the sensors and establishing the connection with sink start during the first phase and the end of communication with sink terminates the second phase.

In the first phase the current is 40.0 mA (with a peak of 60 mA when reading VOC), while in the second phase the current is 21.6 mA. High current consumption in the first phase is due to RGB LED glow, which consumes about 20 mA. Averaging over the time, the power consumption in this mode of operation is 14.5 mA. The total average power consumption is thus 17.2 mA. The total power consumption can be decreased to 13.5 mA, if we keep the LED turned off all the time. If we consider a typical 3.7 V Lithium Polymer (LiPo) battery with a capacity of 2900 mAh, the node can continuously operate for 7 days.

## 6. Conclusions

This paper suggested how to implement an indoor accurate sound-level meter on a low-power and low-cost sensor node. The sound level meter is implemented on the smallest ARM processor available on market—ARM Cortex^TM^-M0. The selection of a small processor core imposed a challenge—how to implement accurate sound-level meter without floating-point unit and with only 4 kB of RAM memory. To address the challenge imposed by small processor we proposed a digital A-weighting filter with quantized coefficients, which has the magnitude response that satisfies tolerance limits imposed by IEC 61672-1. To avoid filter instability, we propose a cascade-form implementation with three second-order sections. Using this method, the A-weighting filtering is performed on the fly (sample-by-sample) and a minimal amount of RAM is used.

The implemented SPL meter has the dynamic range of 72 dB, can reliably sense the sound pressure levels up to 100 dB and is autonomous in its operation. The results of 12-min measurements in the indoor environment showed that the proposed sensor node closely follows values registered by Class 1 Sound Level Meter with the mean difference less than 2 dB. Thus, according to [24], the proposed solution belongs to the Category 3—Low-cost sensor network, which consists of custom-made nodes designed to be inexpensive, low-power and autonomous for large-scale deployments. The proposed sensor node is a promising and viable solution for measurement in application scenarios where legally enforceable sound level data are not needed but measurements are sufficient for informative screening of noise levels.

## Figures and Tables

**Table 1:** Core component list and costs (as of May 2018) for prototype sensor node.

Component	Cost (EUR)
MCU	2.00
pressure sensor	3.00
humidity/temperature sensor	3.30
ambient light sensor	0.50
MEMS microphone	1.15
VOC sensor	20.00
Zigbee module	10.00
3-axes accelerometer	1.00
RGB led	0.50
**Total**	**41.45**

## Abbreviations

MCU	Micro-Controller Unit
WSN	Wireless Sensor Network
SPL	Sound Pressure Level
SLM	Sound Level Meter
RMS	Root Mean Square
ADC	Analog-to-Digital Converter
SOS	Second Order Sections
